# Power Amplifier Predistortion Using Reduced Sampling Rates in the Forward and Feedback Paths

**DOI:** 10.3390/s24113439

**Published:** 2024-05-27

**Authors:** Serien Ahmed, Majid Ahmed, Souheil Bensmida, Oualid Hammi

**Affiliations:** 1 Department of Electrical Engineering, College of Engineering, American University of Sharjah, Sharjah P.O. Box 26666, United Arab Emirates; 2 Institute of Sensors, Signals and Systems, School of Engineering and Physical Sciences, Heriot-Watt University, Edinburg EH14 4AS, UK

**Keywords:** 5G communications, digital predistortion, memory effects, nonlinear distortions, power amplifiers

## Abstract

The feasibility of implementing digital predistortion for next-generation wireless communication is faced with a dilemma due to the ever-increasing demand for faster data rates. This causes the utilized bandwidth to increase significantly, as seen in the 5G NR standard in which bandwidths as high as 400 MHz are utilized. Hence, the development of new predistortion techniques in which the forward and feedback paths operate at lower sampling rates is of utmost importance to realize efficient and practical predistortion solutions. In this work, a novel predistortion technique is presented by which the predistortion is divided between the digital and analog domains. The predistorter is composed of a memoryless AM/AM gain function that is implementable in the analog domain, and a nonlinear model with memory effects in the digital domain to relax the sampling rate requirements on both the forward and feedback paths. Experimental validation was carried out with a 20 MHz and a 40 MHz 5G signal, and the results indicate minimal linearization degradation with a sampling rate reduction of 50% and 30%, respectively. This sampling rate reduction is concurrently applied in the digital-to-analog converter of the forward path and the analog-to-digital converter of the feedback path.

## 1. Introduction

Wireless communications play a vital role in modern societies by enabling a wide range of applications critical for our daily life. Modern communication systems ensure a reliable, seamless, and ubiquitous data transfer that is in the path to connect everyone and everything. This calls for the development of communication systems infrastructure that can handle an increasingly large number of users and an ever-expanding amount of data. Networks that are 5G and beyond offer a plethora of solutions to accommodate these needs in varying applications such as healthcare, inter-vehicular communication, environmental monitoring, and other Internet of things (IoT) applications [1,2,3]. However, these advances can only be achieved through the design of advanced communication hardware that can successfully meet the stringent requirements of modern applications. This work focuses on the radio frequency (RF) front-end of wireless communication systems, and, more specifically, on the power amplification stages where opposing requirements need to be managed carefully.

Radio frequency power amplifiers (PAs) represent one of the most critical subsystems in the RF front-end of modern communication systems. This is mainly due to the noticeable impact that the power efficiency and linearity of the power amplifier have on the overall performance (efficiency and linearity) of the entire transmitter. Power efficiency is an important performance criterion since the power amplification stage is typically the sub-system that consumes the most amount of power in the communication infrastructure (base stations). Enhancing the power efficiency of the PA will reduce the DC power consumption for a given output power, and therefore lessen the amount of power being lost through thermal dissipation. Not only is the unused power being lost, but it often requires the use of cooling mechanisms that will also consume additional power. Hence, power efficiency can have a significant impact on the running cost of base stations. Most importantly, high power consumption increases the carbon footprint of the base station and goes against the need for greener and more environmentally friendly communication infrastructure. Linearity, on the other hand, is directly related to the quality of service and the ability to reliably transmit data without loss of information. Nonlinear distortions, mainly caused by the power amplification stage, cause interference with the adjacent channel and are strictly monitored by spectrum emission regulations in each wireless communication standard. Given that a power amplifier cannot operate in a power efficient mode while linearly amplifying the signal, it is essential, in order to reach an acceptable tradeoff between linearity and efficiency, to operate the power amplifier in a nonlinear but power efficient mode and use adequate system level linearization techniques to counteract the effects of the amplifier’s nonlinear distortions. Among the various linearization techniques, predistortion stands out as the preferred approach for modern base stations [4]. This is mainly due to its ability to achieve adequate performance with a reasonable overhead by taking advantage of digital implementations of the predistortion function.

While digital predistortion (DPD) technique is a mature technology for wireless communication infrastructure, it is facing continuous challenges due to the ever-increasing requirements set by evolving wireless communication standards. For example, compared to 4G systems, 5G systems employ signals of much larger bandwidth that can reach up to 100 MHz in the sub-6 GHz frequency range 1 (FR1), and up to 400 MHz in the millimeter wave frequency range 2 (FR2), as explored in [5,6]. Such wideband signals set tough requirements on the hardware needed for the digital predistortion function (especially for the digital-to-analog and analog-to-digital converters used in predistortion’s forward and feedback paths, respectively). Furthermore, the bandwidth of the signals tends to emulate stronger memory effects in the power amplifier’s behavior. This calls for the development of more advanced predistortion functions that can compensate for such distortions.

Some of the recent work involved with developing new digital predistortion algorithms include developing a bivariate Volterra digital predistorter [7], reduced complexity sequential predistortion [8], MIMO phased arrays focused DPD [9,10,11], developing behavioral models into predistortion models [12], agnostic envelope linearization [13], and a recent paper that delves into the linearization of a 400 MHz bandwidth 5G NR signal [5]. However, one thing that most of the presented works have in common is that they require a feedback signal that is oversampled by as much as five times in order to train the digital predistorters appropriately, such that they account for the adjacent channel spectral regrowth. The work reported in [5], in particular, required a 2 Gsps feedback signal to linearize a 400 MHz signal. Of course, this suggests that to realize a practical DPD solution for signals of wider bandwidths, both the feedback’s analog-to-digital converter (ADC) and forward path’s digital-to-analog converter (DAC) have to accommodate much higher sampling rates. This results in a significant increase in the initial cost of deploying wireless base stations, and it could potentially result in increased power consumption to accommodate the faster data rates, as required with the increased data throughput.

On the other hand, another concern with DPD systems is the computational requirement that increases as the bandwidth increases. As discussed above, the increased intensity of the memory effects that require linearization indicates that more robust DPD algorithms must be used. This comes at the cost of increasing the computational cost of training and utilizing the predistorter. Hence, numerous studies have been conducted in order to alleviate this problem. Some of the proposed solutions include the peak-to-average power reduction techniques presented in [14], the reduced complexity DPD presented in [8], linearization under high data sparsity [15], and the feature selection methodologies presented in [16]. The concept of feature selection [16], in particular, is of great interest, as it attempts to limit the amount of basis functions in a DPD model required for adequate linearization. This utilizes the fact that for many DPD algorithms, most of the defined basis functions contribute insignificantly to linearizing the power amplifier. Hence, eliminating said features, or performing a linear projection of the extracted basis functions into a lower feature space, can help in reducing the computational requirement increase associated with linearizing signals of wider bandwidth. In addition, if this is coupled with relaxing the sampling rates of both the forward and the feedback paths, the practicality of DPD for next-generation wireless communications can be further highlighted. Consequently, accommodating lower sampling rates for both feedback and forward paths is of utmost interest, as it would simplify deploying efficient next-generation wireless infrastructure.

Digital predistortion’s shortcomings when it comes to the feedback and forward paths sampling rates have had some potential solutions suggested in previous research works. However, most research focused on reducing the sampling rates in either the forward or the feedback path separately, which limits their practicality. For instance, the works in [17,18,19] present a novel band-limited feedback approach to reduce the feedback path ADC sampling rate. This was effectively completed by utilizing a bandpass filter prior to the feedback path’s ADC and implementing varying forms of band-limited DPD functions. However, it must be noted that most of the presented work requires additional iterations to create a better estimate of the actual output signal and additional hardware is required to achieve this. Other techniques such as those presented in [20,21,22,23] approach the sampling rates challenges in digital predistortion systems differently by directly under-sampling the feedback signal and allowing some aliasing to occur. The under-sampled feedback signal is then manipulated to cancel out the aliasing effect, and an appropriate DPD model is utilized. Nevertheless, such methods still suffer from similar shortcomings as previously discussed. Furthermore, another approach presented is spectrum stitching [24,25] by which the full bandwidth of the feedback signal is stitched together from several narrow band feedback signals. However, this increases the time required to train a DPD model, as the original feedback signal needs to first be reconstructed. Hence, all of the methodologies presented above focus on relaxing the feedback path’s sampling rate whilst maintaining a higher sampling in the forward path.

On the other hand, to address the sampling requirements of the forward path, RF predistortion [26,27] and hybrid predistortion [28,29] have been suggested. RF predistortion entails applying the predistortion function in the analog domain at radio frequencies. However, implementing RF predistortion requires more hardware components, and due to its analog nature, its flexibility is quite limited when compared to hybrid and digital predistortion counterparts. On the other hand, hybrid predistortion entails utilizing both digital and analog signal processing to implement a predistortion algorithm. An example of that is to have a hybrid two-box model where each box is in a separate domain. This effectively reduces the forward path’s DAC sampling rate requirement, as some of the linearization is implemented in the analog domain. Furthermore, some of the flexibility of DPD is still maintained, as a significant part of the linearization is performed in the digital domain. One example of this is the work presented by Ali et al. in [29]. The predistortion function was divided into two boxes, namely, a memory-based nonlinear box and a memoryless nonlinear box. The main contribution was related to the fact that the memoryless nonlinear function can be implemented in the analog domain to effectively alleviate the linearization effort required in the digital domain. Hence, this allows for utilizing relaxed sampling rates in the forward path as coarse linearization can be obtained by using the analog domain predistorter. This work aims to extend the work proposed in [29] by simultaneously relaxing the sampling rate in both the feedback and the forward paths, and to realize a complete sampling rate reduction.

In this paper, the challenges related to the use of predistortion systems in wideband applications are discussed. It is shown that analog predistortion implementation suffers from poor linearization performance and complex implementation while having the advantage of reducing the sampling rate requirements in the digital-to-analog converter used in the signal’s forward path of digital predistortion systems. Conversely, digital predistortion allows for flexible implementation of the predistortion function and hence is able to reach better linearization performance than the analog predistortion technique. However, this comes at the cost of higher sampling rate requirements on the digital-to-analog converter in the forward path. To achieve a better tradeoff between performance and complexity, a hybrid digital predistortion system is proposed along with reduced sampling rates in the forward and feedback paths. It is shown that the system level architecture, in which the predistortion function is carefully split between the analog and digital domains, allows for relaxed sampling rates in both the forward and feedback paths without compromising the linearization capability of the predistorter. Experimental validation carried on a commercial power amplifier prototype demonstrates the effectiveness of the proposed approach to lead to standard compliant linearization performance while operating at lower sampling rates in the signal forward and feedback paths.

The main contribution of this paper is related to the development of a predistortion system in which reduced sampling rates are used in both the forward path and the feedback path, hence, making the ground work for the development of high performance predistortion systems suitable for 5G applications and beyond.

Section 2 discusses the challenges related to predistortion systems design with a focus on the sampling rate requirements in the signal forward and feedback paths. The proposed hybrid digital/analog predistortion system is thoroughly discussed in Section 3. The experimental setup and test results are presented in Section 4. The conclusions are summarized in Section 5.

## 2. On the Challenges of Predistortion Systems Design

Predistortion relies on applying a complementary nonlinear function prior to the nonlinear amplifier, such that the cascade of both nonlinear functions is equivalent to a linear one. The amplifier’s complementary nonlinear function is commonly implemented either in digital domain (as it is the case in digital predistortion techniques), or in the analog domain (as it is the case in analog predistortion techniques). The linearization performance of predistortion systems heavily relies on two key aspects, which are the accurate characterization of the amplifier’s nonlinear behavior and the precise synthesis of the desired predistortion function that will perfectly complement the nonlinear behavior of the power amplifier. Despite being considered a mature technology, predistortion systems require continuous efforts to enhance their performance and cope with the evolution of wireless communication standards. In [4], a thorough study of predistortion systems and their challenges is presented with an emphasis on emerging 5G and beyond applications especially at millimeter waves.

**Figure 1:**
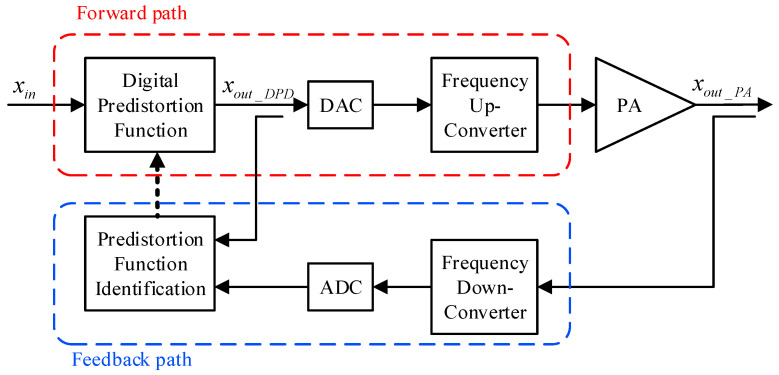
Simplified functional block-diagram of a digital predistortion system.

**Figure 2:**
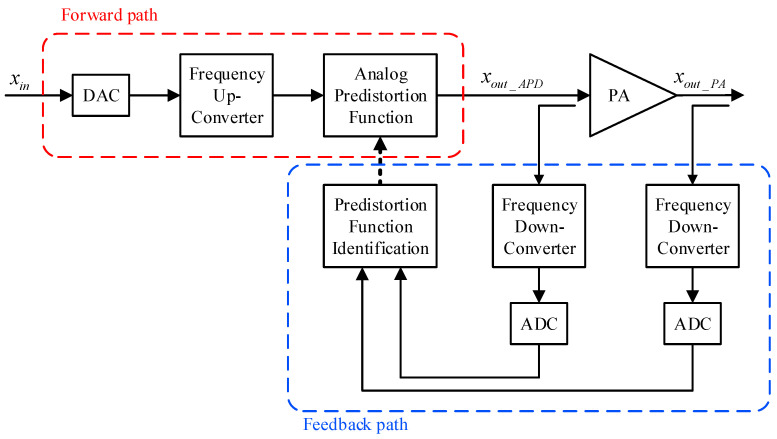
Simplified functional block-diagram of an analog predistortion system.

A simplified functional block diagram of a digital predistortion system is reported in Figure 1. Conversely, Figure 2 depicts a simplified block diagram of an analog predistortion system. In the digital predistortion system of Figure 1, since the signal is predistorted in the digital domain, the DAC is being fed with the signal $x_{out\_DPD}$. This predistorted signal typically has a bandwidth that is five times that of the input signal $\left(x_{in}\right)$ due to the nonlinear distortions generated by the predistortion function. Therefore, the DAC needs to be able to handle such a bandwidth. Similarly, the signal at the output of the amplifier $\left(x_{out\_PA}\right)$ is also expected to have a bandwidth that is five times that of the input signal to capture the full extent of the spectral regrowth caused by nonlinear distortions. Hence, the ADC in the feedback path needs to be able to handle such a bandwidth. These constraints on the bandwidth of the signals being fed to the digital-to-analog and the analog-to-digital converters in the forward and the feedback paths, respectively, represent a bottleneck for digital predistortion systems, especially in the case of wideband signals. In digital predistortion systems, the signals used for the predistortion function identification are the predistorted signal $x_{out\_DPD}$ (already in the digital domain) and the amplifier’s output signal $x_{out\_PA}$ which is in the analog RF domain. Hence, only one feedback path is needed in order to down-convert and digitize the amplifier’s output signal. An inherent advantage of this approach relies on the fact that the signals used for the predistortion function identification include the power amplifier nonlinearities as well as any possible impairments caused by the frequency up-conversion stage.

A closer look at the block diagram of Figure 2 shows that in analog predistortion systems, the signal at the input of the DAC in the forward path is the original signal and not the predistorted one. This alleviates the requirements on the DAC sampling rate in the forward path. Conversely, the signal at the output of the amplifier still requires a sampling rate commensurate to five times the bandwidth of the input signal. Therefore, the ADC sampling rate requirements in the feedback path of analog predistortion systems is identical to that of digital predistortion systems. Moreover, since the predistortion function is applied just before the power amplifier, the signals used for the identification of the predistortion function, that is, the predistorted signal $\left(x_{out\_APD}\right)$ and the amplifier’s output signal $\left(x_{out\_PA}\right)$, are both in the analog RF domain. Hence, two frequency down-conversion and digitization paths are needed for the feedback path. Moreover, using the signals $x_{out\_APD}$ and $x_{out\_PA}$ for the identification of the predistortion function does not capture any impairments that occur within the forward path’s frequency up-conversion stage. This limitation is compounded with the need for two frequency down-conversion stages and two analog-to-digital converters, which constitutes a major complexity overhead in analog predistortion systems when compared to digital predistortion systems. Accordingly, with respect to the accurate characterization of the power amplifier behavior, the analog predistortion architecture presents a single advantage related to the relaxed sampling rate requirements in the DAC of the forward path. However, this comes at the cost of an additional down-conversion and digitization block in the feedback path.

A key difference between digital predistortion and analog predistortion systems is related to the implementation of the predistortion function. For digital domain implementation, a plethora of models exist and can be used to accurately synthesize a predistortion function that will lead to a quasi-perfect match between the nonlinear characteristics of the DPD and that of the power amplifier, hence, ensuring optimal linearization performance and spectrum regrowth cancellation. However, the use of analog implementation of the predistortion function significantly limits the options available. In fact, analog predistortion mainly uses analog nonlinear devices/circuits that operate as distortion generators or a very limited set of analytically defined functions such as look-up tables, and the envelope memory polynomial. This results in reduced linearization performance when compared to digital predistortion systems, especially in the case of highly nonlinear power amplifiers exhibiting memory effects. Accordingly, digital predistortion offers a significant advantage in terms of performance.

In addition to its ability to synthesize accurate predistortion profiles, digital predistortion functions are easy to implement through an update of their coefficients and parameters. On the other hand, the update of the analog predistortion functions is very tedious and does not offer the same degree of flexibility observed in digital predistortion systems.

From the discussion above, it appears that digital predistortion as well as analog predistortion systems each suffer from several key limitations, as summarized in Table 1. These limitations are at the origin of the motivation behind this work in which a hybrid predistortion function that allies the advantages of digital and analog systems is proposed. As summarized in Table 1, the proposed hybrid predistorter which employs a digital predistortion function and an analog predistortion function aims at taking advantage of the reduced sampling rate in the forward path, which is characteristic of analog predistortion systems, while avoiding the limitations associated with the analog implementation by augmenting it with a digital predistortion function. The digital predistortion function enables the use of high accuracy adaptive predistortion functions.

## 3. Proposed Predistortion System

### 3.1. Proposed Predistortion System Description

**Figure 3:**
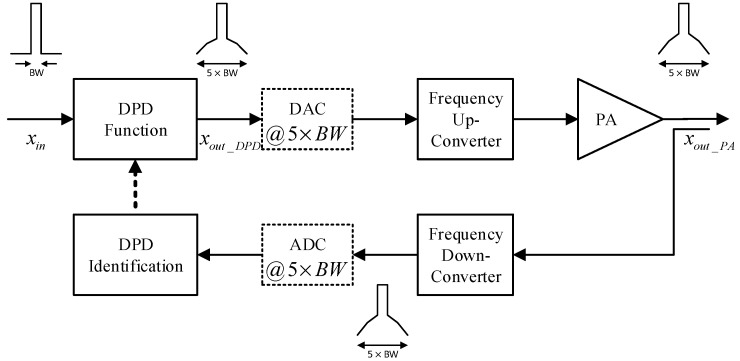
Generic digital predistortion system architecture detailing signal bandwidths.

**Figure 4:**
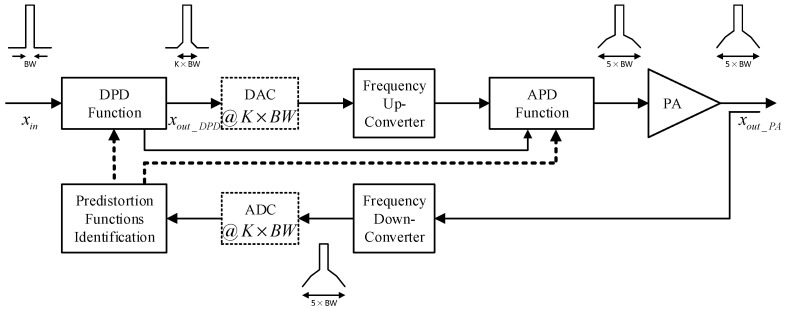
Proposed hybrid digital/analog predistortion system architecture detailing signal bandwidths.

In this work, a hybrid digital/analog predistortion system is considered. The proposed system builds upon conventional digital predistortion systems (such as the one reported in Figure 3) by adding a second predistortion function in the analog domain, as illustrated in the block diagram of Figure 4. Comparing the block diagrams of Figure 3 and that of Figure 4, it appears that adding a predistortion function in the analog domain leads to several benefits that make the hybrid predistortion system superior to its fully digital and fully analog counterparts. These competitive advantages will be discussed in this section and corroborated with the experimental results derived in the next section.

Typical power amplifiers nonlinearity results in spectral regrowth that spans over five times the bandwidth of the input signal. Predistortion techniques that are used to linearize such amplifiers will result in a similar (in terms of bandwidth) spectral regrowth. Therefore, in common predistortion systems (such as the one depicted in Figure 3), the signals $x_{out\_DPD}$ and $x_{out\_PA}$ are expected to have five times the bandwidth of the input signal $x_{in}$. Hence, the forward path’s DAC and the feedback path’s ADC are expected to operate at a sufficiently high sampling rate to accommodate signals with a bandwidth equal to 5×BW, where BW refers to the bandwidth of the input signal. Several solutions have been proposed to relax the sampling rate of the ADC used in the feedback path [18,19,20,21,22,23,24,25]. This is possible since the in-band frequency range includes information about higher order distortions [8]. However, to the best of the author’s knowledge, there was no reported attempt to simultaneously reduce the sampling rates of the forward path’s DAC and the ADC used in the feedback path.

In the proposed hybrid predistorter, this major limitation of digital predistortion systems is addressed by adding a second predistortion function in the analog domain. The analog predistortion function’s main purpose is to perform a coarse linearization of the device under test with a focus on the strongly nonlinear memoryless distortions. The analog predistortion function is optimized for simplicity and will be made of a simple memoryless look-up table (LUT), hence avoiding the implementation complexity typically associated with analog predistorters. The analog predistortion function is computed by the predistortion functions identification block which is responsible of synthesizing the look-up table of the analog predistorter (APD) and the memory polynomial function of the DPD. The parameters computed by the predistortion function’s identification block are fed to the two predistortion functions. Moreover, since the analog predistorter will perform a coarse compensation of the highly nonlinear memoryless distortions of the PA, the cascade made of the APD, and the PA will behave as a nonlinear system with mild nonlinearity. However, the memory effects exhibited by the PA will also be observed since they are not compensated for using the APD. Hence, the digital predistortion function will be almost linear as it will be mainly compensating for the mildly nonlinear residual distortions that remain following the application of the analog predistortion function.

The analog predistortion function preferred implementations include several options such as the use of an RF vector multiplier, a variable gain amplifier, or a variable attenuator. The update of the analog predistortion function refers to the update of the look-up table function and not the hardware of the analog predistorter itself. In fact, the update is applied to the mapping function that translates the output of the digital predistorter into the control signals that need to be applied to the analog predistortion function to result in the desired APD gain. This mapping is a function of the transfer characteristics of the analog predistorter used. Moreover, in all cases, the analog predistortion function is subject to imperfections associated with analog domain implementations. These can include delay misalignment between the control signal and the RF signal, as well as limited resolution and accuracy in the implementation of the gain values. These issues are commonly observed in analog predistorters. The use of a second predistortion function, which will be applied to linearize the cascade made of analog predistorter and the device under test, is expected to partially compensate for such imperfections. It is important to note here that these imperfections are observed in analog predistorters in general and are not specific to the proposed predistorter.

The digital predistortion function is derived from the signals $x_{out\_DPD}$ and $x_{out\_PA}$, yet, it is performed when the APD function is being applied. Hence, the $x_{out\_DPD}$ and $x_{out\_PA}$ will be the input and output waveforms of a mildly nonlinear system. Since the nonlinearity order of the system being linearized by the DPD function is less than that of the PA alone, the spectral regrowth caused by the DPD function in the case of the proposed hybrid predistortion system will be less than that observed in the case of the conventional digital predistortion system. Consequently, it is anticipated that the bandwidth of the signal at the output of the DPD will be K×BW with K<5. This will result in a sampling rate reduction for the DAC used in the signal’s forward path. Accordingly, the architecture of the proposed hybrid predistorter is suitable for reducing the sampling rate in the forward and feedback paths through the use of the analog predistortion function in conjunction with the digital predistortion function. Moreover, the procedure used to identify the two predistortion functions lessens the accuracy requirements on the APD function, and hence reduces its implementation complexity. Here, it is important to note that the reduction in the sampling rate of the forward path’s DAC and that of the feedback path’s ADC can be independent. However, from a computational complexity perspective, it is more convenient to operate the two converters at the same sampling rates to ensure that the two signals used for the predistortion function identification have identical sampling rates and to avoid the digital signal processing overhead associated with re-sampling one signal to match the other signal’s sampling rate for time-delay alignment and digital predistortion function identification.

### 3.2. Identification Process of the Proposed Predistortion System

**Figure 5:**
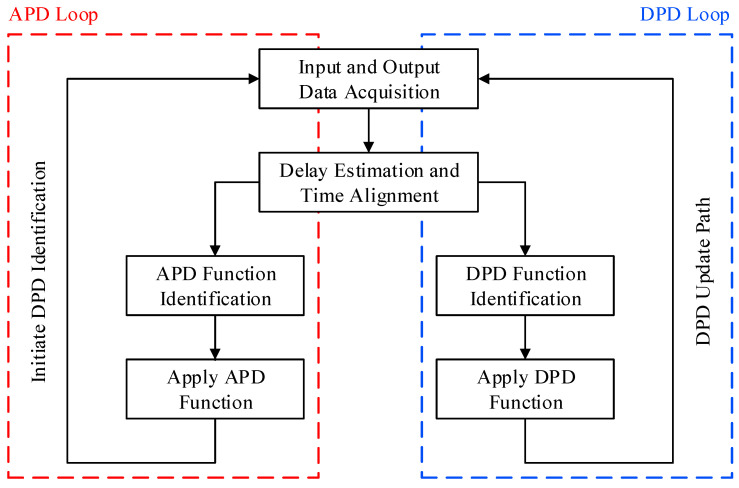
Flow chart of the proposed hybrid DPD system identification and update.

The flow chart of the proposed hybrid predistortion system is presented in Figure 5. First, the input and output waveforms to be used for the power amplifier characterization are acquired. The input waveform corresponds to the signal at the output of the DPD block $\left(x_{out\_DPD}\right)$ while the output waveforms refers to the amplifier’s output signal $\left(x_{out\_PA}\right)$. Initially, the APD and DPD functions are bypassed, that is, their outputs are equal to their inputs. During this first characterization step, the forward path’s DAC and feedback path’s ADC operate at a sampling rate that is commensurate to K×BW, where BW represents the input signal’s bandwidth, and K is a factor such that K<5. The delay between the two waveforms is estimated and compensated for. Then, the analog predistortion function is identified.

The analog predistortion function used in this work is a memoryless look-up table such that

(1) $$x_{out\_APD}\left(n\right)=x_{in\_APD}\left(n\right)\cdot G\left(\left|x_{in\_APD}\left(n\right)\right|\right),$$

where $x_{in\_APD}$ represents the input waveform of the APD’s input signal, and $G\left(\left|x_{in\_APD}\right|\right)$ is the memoryless gain function of the APD. Typically, look-up-table-based predistorters are complex-valued, in the sense that the gain $G\left(\left|x_{in\_APD}\right|\right)$ has a magnitude and phase that are used to compensate for the amplitude modulation to amplitude modulation (AM/AM) and amplitude modulation to phase modulation (AM/PM) distortions of the PA, respectively. The AM/AM characteristic is obtained by computing the magnitude of the instantaneous gain of the device under test as a function of the instantaneous input power. The AM/PM characteristic corresponds to the phase of the instantaneous gain of the device under test as a function of the instantaneous input power.

In the proposed system, the APD function is built using a real-valued memoryless look-up-table-based APD which only compensates for the AM/AM distortions. This function is obtained from the measured input and output waveforms using an exponentially weighted moving average algorithm [30]. The AM/PM distortions compensation is performed within the digital predistortion function. The rationale behind this design choice is twofold. First, the use of a real-valued LUT that compensates for memoryless AM/AM distortions only will reduce the implementation complexity of the APD function. Second, most of the power amplifiers employed in modern base stations exhibit mildly nonlinear AM/PM distortions that are mainly dominated by memory effects. Thus, AM/PM compensation can be embedded within the digital predistortion function only.

The APD function is synthesized as a look-up table containing real-valued power-dependent gain values. This function can be implemented using a variable gain amplifier, a voltage-controlled attenuator, or using an RF vector multiplier. Such implementations are preferred since they allow for the control of the nonlinearity profile in contrast with analog predistortion functions where the nonlinearity is implemented using diodes and intermodulation distortion generators. In this paper, the APD is emulated by applying its transfer characteristics in MATLAB (release R2022B) using a look-up table model.

Following the identification of the APD function, this latter is applied to linearize the power amplifier. Therefore, the distortions as well as the spectral regrowth at the output of the power amplifier will be reduced during the second characterization step that will be used to derive the DPD function. Consequently, the impact of the low sampling rate in the feedback path will be reduced since the bandwidth of the signal at the output of the amplifier is narrower following the partial linearization performed by the APD function. Moreover, since the characterization data used to derive the DPD function includes the cascade made of the APD and the power amplifier, two important benefits can be achieved. First, the DPD will be able to compensate for residual distortions due to the imperfections and inaccuracy of the APD function as well as the amplifier’s memory effects. Second, because the cascade made of the APD and the power amplifier is equivalent to a mildly nonlinear system, the DPD function will be mildly nonlinear and hence, results in a diminished spectral regrowth at the output of the predistorter, as illustrated in the sketched spectrum of the signal $x_{out\_DPD}$ in Figure 4. Therefore, it is possible to use reduced sampling rate in the signal’s forward path DAC even when the digital predistortion function is applied.

In the proposed hybrid predistorter, the digital predistortion function is implemented using a memory polynomial model. Hence, the output waveform of the digital predistorter is related to its input through the following:

(2) $$x_{out\_DPD}\left(n\right)=\sum_{i=1}^{N}\sum_{m=0}^{M}a_{im}x_{in\_DPD}\left(n-m\right)\cdot {\left|x_{in\_DPD}\left(n-m\right)\right|}^{i-1},$$

where $x_{in\_DPD}$ and $x_{out\_DPD}$ are the baseband complex waveforms at the input and output of the digital predistortion functions, respectively. N and M represent the nonlinearity order and the memory depth of the memory polynomial function, respectively. $a_{im}$ represents the complex-valued coefficients of the memory polynomial function. For a given nonlinearity order (N) and memory depth (M), the total number of coeffcieints in the memory polynomial function is given by N×(M+1).

When written in a matrix form, Equation (2) becomes

(3) $$x_{out\_DPD}\left(n\right)=X_{in\_DPD}\left(n\right)\cdot A,$$

where

(4) $$A={\left[\begin{array}{ccc} a_{10} & a_{11} & a_{12}\;\cdots \begin{array}{cc} \;a_{1m} & a_{20}\;\cdots \;\begin{array}{cc} a_{N0} & a_{NM} \end{array} \end{array} \end{array}\right]}^{T},$$

and

(5) $$X_{in\_DPD}\left(n\right)=\left[\begin{array}{cccc} X_{in\_DPD,1}\left(n\right) & X_{in\_DPD,2}\left(n\right) & \cdots & X_{in\_DPD,N}\left(n\right) \end{array}\right],$$

with

(6) $$X_{in\_DPD,i}\left(n\right)=\left[\begin{array}{ccc} x_{in\_DPD}\left(n\right){\left|x_{in\_DPD}\left(n\right)\right|}^{i-1} & \cdots & x_{in\_DPD}\left(n-M\right){\left|x_{in\_DPD}\left(n-M\right)\right|}^{i-1} \end{array}\right].$$

In these equations, ${\left[\right]}^{T}$ refers to the transpose function.

The identification of the memory polynomial coefficient is performed by solving the linear system obtained when considering the *L* samples of the waveforms used to identify the coefficients of the polynomial model. In this work, 8000 samples were used to compute the polynomial coefficients using the Moore–Penrose pseudoinverse technique.

Accordingly,

(7) $$A=pinv\left(X_{in\_DPD}\right)\cdot X_{out\_DPD}.$$

In Equation (7), $pinv\left(X_{in\_DPD}\right)$ is given by

(8) $$pinv\left(X_{in\_DPD}\right)={\left[{\left(X_{in\_DPD}\right)}^{T}X_{in\_DPD}\right]}^{-1}\cdot {\left(X_{in\_DPD}\right)}^{T},$$

where

(9) $$X_{in\_DPD}={\left[\begin{array}{cccc} X_{in\_DPD}\left(s\right) & X_{in\_DPD}\left(s+1\right) & \cdots & X_{in\_DPD}\left(s+L-1\right) \end{array}\right]}^{T},$$

and

(10) $$X_{out\_DPD}={\left[\begin{array}{cccc} x_{out\_DPD}\left(s\right) & x_{out\_DPD}\left(s+1\right) & \cdots & x_{out\_DPD}\left(s+L-1\right) \end{array}\right]}^{T}.$$

s refers to the starting index of the signal samples used for the coefficients identification.

It is worth noting that the identification of both predistortion functions is performed using the indirect learning technique where the predistortion function is directly synthesized from the measured data without the need to derive a model of the device under test. Moreover, the digital predistortion function can be implemented either as a memory polynomial or any other function that compensates for memory effects such as the Volterra series. However, the digital predistortion function aims mainly at compensating the nonlinear distortions of the cascade made of the APD and the device under test, and this cascade behaves as a mildly nonlinear system with memory effects. Hence, a memory polynomial function seems adequate when compared to more complex functions such as the Volterra series, which would result in comparable performance at a much higher computational cost. However, Volterra series can be considered for cases where the nonlinear distortions of the cascade made of the APD and DUT are too complex to be adequately compensated for using a memory polynomial function.

## 4. Experimental Validation

In this section, the experimental setup used in this work is first presented along with the procedure followed during the experimental validation. Two case studies are then reported. In the first case study, the proposed predistorter is validated using a 20 MHz test signal. In the second case study, the performance of the proposed predistorter is evaluated using a 40 MHz test signal. In each case study, the performance of the proposed predistorter is assessed for several sampling rates in the forward and feedback paths and is benchmarked against state of the art models.

### 4.1. Device under Test and Experimental Setup

**Figure 6:**
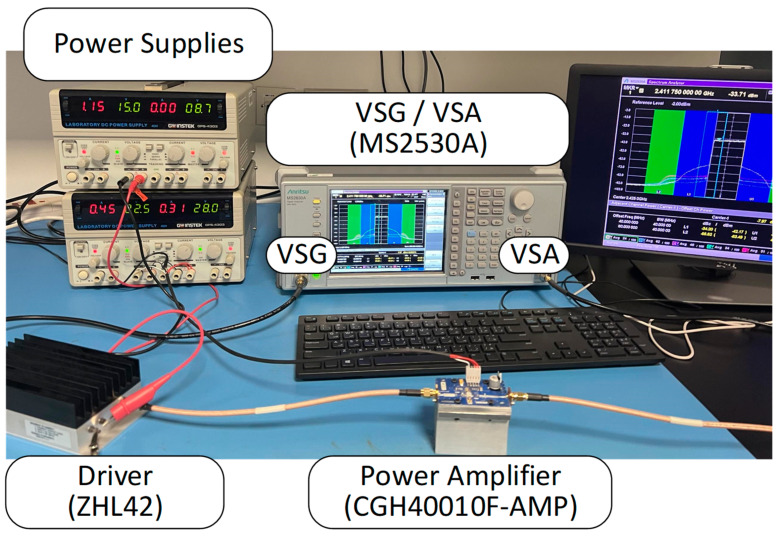
Photograph of the experimental setup.

Figure 6 shows a photograph of the experimental setup. The MS2530A test instrument, from Anritsu, Kanagawa, Japan is used as a vector signal generator (VSG) and a vector signal analyzer (VSA). The baseband digital waveform is synthesized in a computer where all digital signal processing is performed. The baseband waveform is downloaded into the vector signal generator and the corresponding RF signal is produced. The RF signal is applied to the driver (ZHL 42 from Mini-Circuits) in order to increase its power to be able to drive the power amplifier into its nonlinear region. The signal at the output of the power amplifier is attenuated and then fed into the VSA. The signal down-conversion and digital demodulation are performed within the VSA, and the resulting complex baseband waveform is then saved into the computer for further processing (time-delay estimation and alignment, and predistortion functions identification).

The test instrument used in this work has a maximum sampling rate of 120 MSa/s in the signal generator part, and 200 MSa/s in the signal analysis part. These correspond to a maximum vector modulation bandwidth of 120 MHz, and a maximum signal analysis bandwidth of 125 MHz. This sets an upper limit on the bandwidth of the test signals that can be used for experimental validation.

The power amplifier used in this work is a 10 W class AB commercial gallium nitride (GaN) power amplifier (model CGH40010F-AMP from Wolfspeed, Durham, NC, USA). The amplifier is designed to operate in the sub-6 GHz frequency band. During the experiments, the amplifier was operated around a carrier frequency of 2425 MHz using two 5G test signals. The two test signals used in this work were both generated according to the 5G new radio (NR) standard. The first signal has a bandwidth of 20 MHz, while the second one has a bandwidth of 40 MHz. The peak to average power ratios of these signals are 10.7 dB and 10.9 dB, respectively. During the tests, including characterization and performance assessment of the proposed predistorter, the power levels were set such that the peak power at the output of the power amplifier is 42 dBm, which corresponds to the saturation power of the CHG40010F-AMP device from Wolfspeed, Duham, NC, USA.

**Figure 7:**
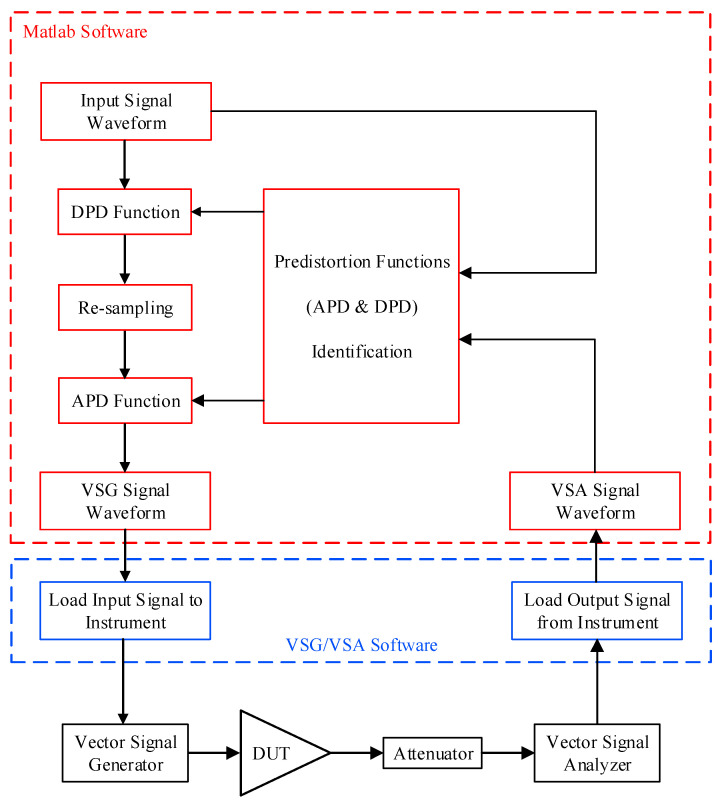
Simplified functional block diagram of the experimental setup.

A functional block diagram of the experimental setup is presented in Figure 7. For the experimental validation performed in this work, the DPD and APD functions were implemented in MATLAB Software. During the first step of the process (referred to as APD loop in Figure 5), in which the APD function is identified, the DPD function, the re-sampling block, and the APD are bypassed. The input signal waveform is generated at the reduced sampling rate specified in the test case, as will be described in the next subsection. The output of the power amplifier measured under this condition is also acquired at the reduced sampling rate and is used along with the input signal to identify the APD function. In the second step, referred to as DPD loop in the flow chart of Figure 5, the DPD function is bypassed, and the input signal waveform generated at the reduced sampling rate is fed into the re-sampling block, which will increase the sampling rate to its highest value. This signal is then applied into the APD and downloaded into the vector signal generator. The corresponding RF signal is applied to the device under test, and the partially linearized output is acquired at the reduced sampling rate. The input signal waveform and the VSA signal waveform are then used in the predistortion functions identification block to derive the DPD function.

Figure 7 shows that the input signal waveform is first fed into the DPD function. A re-sampling block is placed at the output of the DPD function. It is important to note that the signal re-sampling block is not needed for the implementation of the proposed predistorter. It is only used in this experimental validation to allow the DPD function and the APD function to operate at different sampling rates. This allows for including the effect of reduced sampling rate in the forward path while operating the APD at a higher sampling rate. The re-sampling block will produce a signal sampled at a sufficiently high rate to allow for the presence of all inter-modulation distortions and spectrum regrowth that will be caused by the APD function. The signal at the output of the re-sampling block is applied to the APD function. In here, the APD function is simulated using an AM/AM look-up table. The signal at the output of the APD is saved and downloaded into the vector signal generator. The VSG generates the corresponding RF signal that will be applied to the device under test made of the driver and the power amplifier. This allows for the realistic evaluation of the predistortion system performance while having a power amplifier prototype in the loop. The signal at the output of the power amplifier is fed into the vector signal analyzer to compute the adjacent channel leakage ratio and assess the performance of the predistortion system and its effectiveness in reducing the spectrum regrowth.

**Figure 8:**
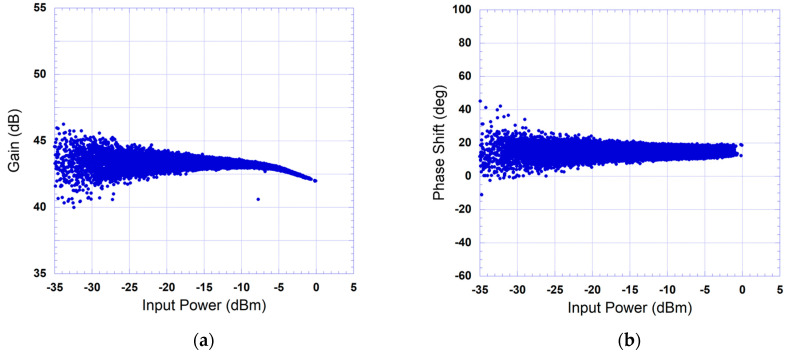
Measured instantaneous gain characteristic of the device under test when driven by a 5G test signal with 20 MHz bandwidth: (**a**) AM/AM characteristic; (**b**) AM/PM characteristic.

**Figure 9:**
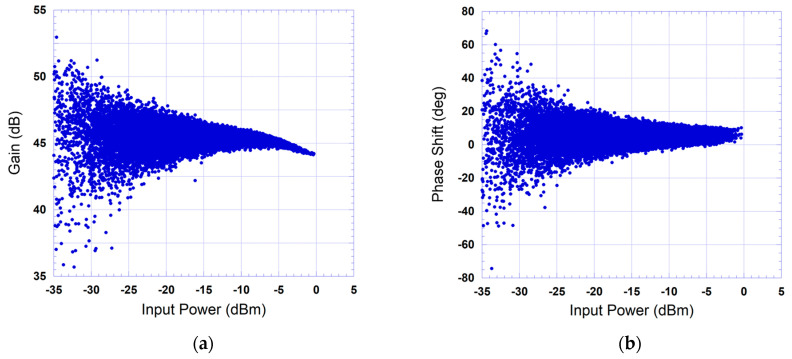
Measured instantaneous gain characteristic of the device under test when driven by a 5G test signal with 40 MHz bandwidth: (**a**) AM/AM characteristic; (**b**) AM/PM characteristic.

The measured complex gain characteristics of the device under test are reported in Figure 8 and Figure 9 for the 20 MHz and the 40 MHz test signals, respectively. The device under test here refers to the cascade made of the driver and the power amplifier. The AM/AM characteristic depicted in Figure 8a shows that the power amplifier has a nonlinear behavior with mild memory effects as illustrated in the spread of the data points. Conversely, the AM/PM characteristic of Figure 8b is mainly dominated by the dispersion due to memory effects and does not manifest any significant nonlinearity. The measured gain characteristics corresponding to the 40 MHz test signal show similar nonlinear behavior to what was observed for the 20 MHz test signal but with a noticeably stronger memory effects, as demonstrated by the wide spread of the data points in Figure 9.

The experimental validation of the proposed predistorter was performed using each of these two test signals. For each signal, the proposed predistorter was derived while operating the forward path’s digital-to-analog converter and the feedback path’s analog-to-digital converter at consistent sampling rates. These sampling rates were decreased iteratively and the performance of the proposed predistorter was recorded for the different sampling rates. In order to compare the achieved results to state of the art predistorters, the reverse twin-nonlinear two-box (RTNTB) [31] predistorter and the envelope memory polynomial (EMP) [32] predistorters were used as benchmark. The rationale behind the selection of these models for the comparative analysis is mainly attributed to their relative similarity to the proposed predistorter. In fact, the reverse twin-nonlinear two-box model is a two-box predistorter that uses a memory polynomial function followed by a look-up table. The two differences between the RTNTB predistorter and the proposed hybrid predistorter are related to the type of look-up table sub-function. First, in the reverse twin-nonlinear two-box predistorter, the look-up table is complex-valued and compensates for both the memoryless AM/AM distortions and the memoryless AM/PM distortions. Conversely, in the proposed predistorter, the look-up table sub-function is real-valued and compensates only for the memoryless AM/AM distortions. Second, in the RTNTB model, the look-up table sub-function is implemented digitally while in the proposed predistorter the look-up table sub-function is intended for analog implementation. The envelope memory polynomial is selected as a second benchmark DPD since it allows for the compensation of memory effects while being suitable for analog implementation. Hence, the twin-nonlinear two-box model represents a best-case scenario in terms of performance of a fully digital predistorter, and the envelope memory polynomial model represents a best-case scenario in terms of performance of a potential analog predistortion function that can compensate for memory effects.

**Figure 10:**
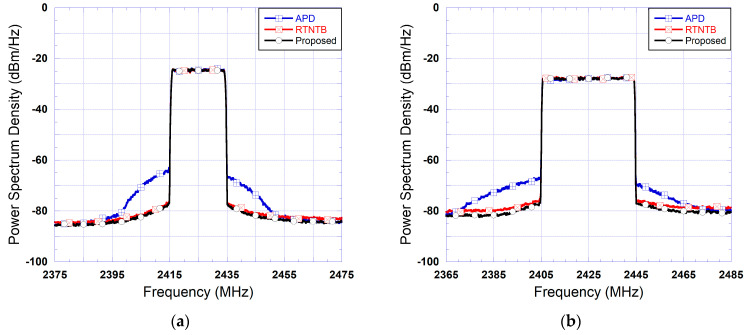
Measured spectra at the output of the device under test when linearized using the proposed predistorter, a digital predistorter, and an analog predistorter: (**a**) Case of the 20 MHz test signal; (**b**) Case of the 40 MHz test signal.

To assess the performance of the proposed predistorter architecture, Figure 10 presents the spectra measured at the output of the power amplifier when linearized using the proposed predistorter, the benchmark digital predistorter (using the RTNTB structure), and the analog predistorter (using a complex-valued look-up table structure). As can be seen from this figure, the analog predistorter performance is quite limited. This is mainly due to its inability to compensate for the memory effects of the device under test. Furthermore, the performance of the proposed predistorter is identical to that of the RTNTB benchmark. Table 2 includes the measured ACLR at the output of the device under test for the test conditions reported in Figure 10. In this table, and throughout the paper, ACLR_L refers to the lower channel(s) ACLR (corresponding to a negative offset frequency) while ACLR_U refers to the upper channel(s) ACLR (corresponding to a positive offset frequency). These ACLR values are consistent with the results observed in the frequency domain and show that the proposed predistorter consistently outperforms the benchmark DPD by a slight margin.

**Figure 11:**
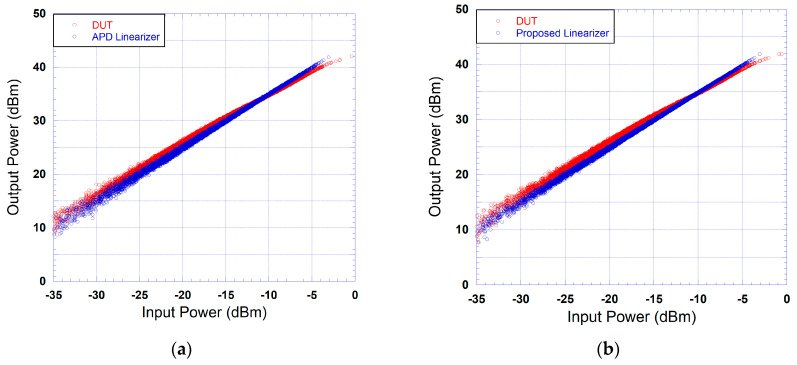
Measured output power versus input power characteristics of the DUT alone and the cascade made of the linearizer and DUT: (**a**) APD linearizer; (**b**) proposed linearizer.

**Figure 12:**
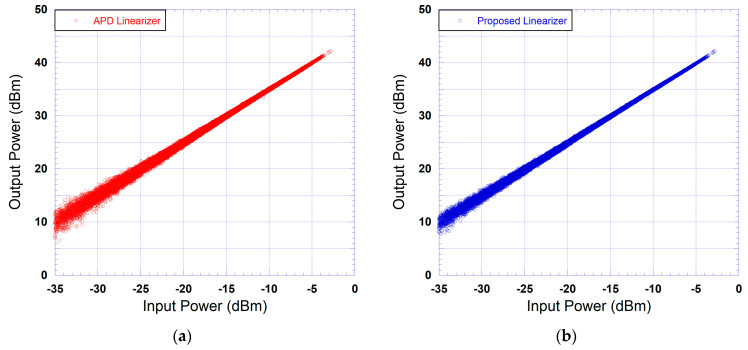
Measured output power versus input power characteristics of the cascade made of the linearizer and DUT: (**a**) APD linearizer; (**b**) proposed linearizer.

The output power versus input power characteristics of the device under test and that of the cascade made of the device under test and the predistorter are reported in Figure 11 for the case where an APD is used and the case where the proposed predistorter is used. These curves clearly show the ability of the proposed predistorter to linearize the transfer characteristic of the device under test. For the case of the analog predistorter, a similar improvement in the linearity of the output power versus input power characteristics is obtained. In order to compare the linearization performance of the proposed predistorter with respect to that of the APD, the output power versus input characteristic of the case made of the linearizer and the DUT is reported in Figure 12 for the case of the APD and that of the proposed DPD. As it appears through this figure, both linearizers compensate for the nonlinearity of the output power versus input power characteristic. However, in the case of the APD, the residual dispersion observed is significantly higher than that observed in the case of the proposed predistorter. This is mainly due to the fact that the APD does not compensate for the memory effects of the DUT, and hence, significant dispersion in the output power characteristic remains.

### 4.2. Case I—Validation Using 20 MHz Test Signal

**Figure 13:**
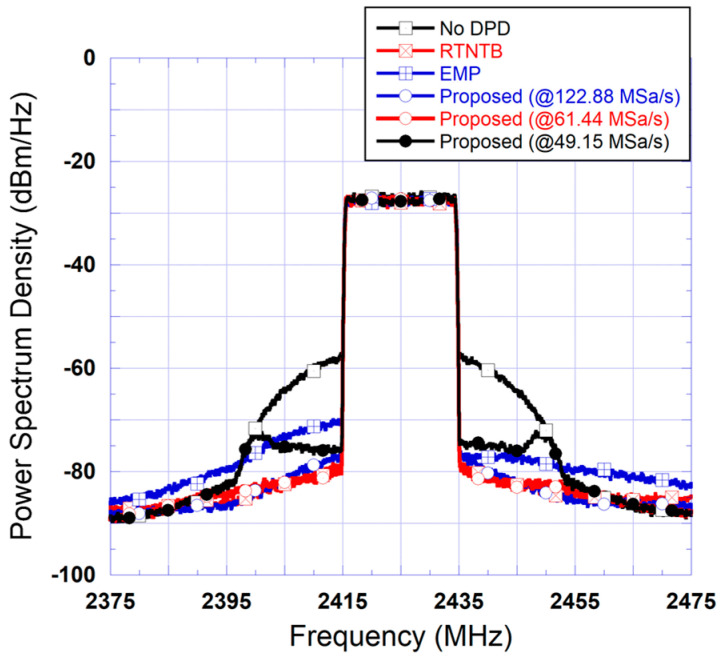
Measured spectra at the output of the linearized amplifier using benchmark digital predistorters and the proposed predistorter derived and implemented using various reduced sampling rates (Case I: 20 MHz test signal).

In the first test, the 5G signal having a 20 MHz bandwidth was used to assess the performances of the proposed predistorter as well as the two benchmark predistorters. Initially, the digital-to-analog converter in the forward path and the analog-to-digital converter in the feedback path were operated at a sampling rate of 122.88 MSa/s. The device under test was first linearized using the proposed hybrid predistorter as well as the RTNTB predistorter and the EMP predistorter using the reference sampling rate of 122.88 MSa/s. Then, the sampling rates used in the forward and feedback paths were reduced successively to 61.44 MSa/s and 49.15 MSa/s. These correspond to a reduction of 50% and 60% when compared to the reference sampling rate, respectively. The spectra measured at the output of the power amplifier using these five predistorters as well as the spectra measured before linearization are reported in Figure 13. This figure shows that before linearization, the power amplifier had significant distortions. These distortions are reduced by approximately 20 dB when the RTNTB predistorter is used to linearize the amplifier. Similar results are obtained with the proposed predistorter when a sampling rate of 122.88 MSa/s is used in the forward and feedback paths. Conversely, the EMP-based predistorter, even though derived from 122.88 MSa/s sampling rate, leads to noticeable residual nonlinear distortions in the adjacent channels, hence, clearly depicting the limits of this predistorter. Figure 13 also shows the spectra measured at the output of the power amplifier when the proposed predistorter is derived and applied using the 61.44 MSa/s and 49.15 MSa/s sampling rates. These results show that reducing the sampling rate in the forward and the feedback paths by 50% from 122.88 MSa/s to 61.44 MSa/s does not have any noticeable effect on the proposed predistorter’s performance. However, a further reduction in the sampling rate to 49.15 MSa/s clearly impacts the performance of the predistorter and its ability to reduce spectrum regrowth.

The results reported in Figure 13 were derived for the case where the memory polynomial function in the RTNTB predistorter as well as the proposed predistorter has a total of 15 coefficients (that is three branches with a fifth order nonlinearity in each). Here, it is important to note that the coefficients of the memory polynomial function in these two DPDs are identified independently. Moreover, the EMP results reported are also for the same nonlinearity order and memory depth.

**Figure 14:**
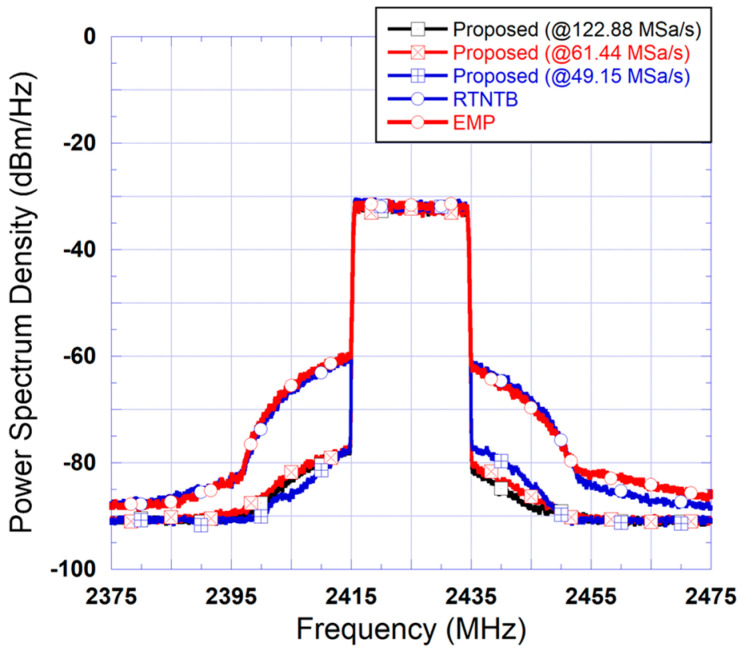
Measured spectra at the input of the digital-to-analog converter in the signal forward path for the benchmark digital predistorters and the proposed predistorter derived and implemented using various reduced sampling rates (Case I: 20 MHz test signal).

To illustrate the advantage of the proposed predistorter in relaxing the sampling rate of the digital-to-analog converter in the signal forward path, the signals expected at the input of the DAC of the forward path in each of the three considered predistorters were observed. Figure 14 includes the spectra of the signals at the input of the DAC used in the forward path for the two benchmark predistorters as well as the three versions of the proposed predistorter (derived using sampling rates of 122.88 MSa/s, 61.44 MSa/s, and 49.15 MSa/s). The results shown in this figure clearly demonstrate that for the proposed predistorter, the signals present at the input of the DAC have much narrower bandwidth and therefore can be accommodated with reduced sampling rates without significant loss of performance. It also shows that for the proposed predistorter versions corresponding to sampling rates of 122.88 MSa/s and 61.44MSa/s, the signals are almost identical. However, the spectrum of the signal corresponding to 49.15 MSa/s rate deviates slightly from these, hence, causing a loss of linearization performance, as observed in the amplifier’s output spectra of Figure 13.

To have a better insight about the relative performance of the various predistorters, the size of the memory polynomial function was varied by sweeping the nonlinearity order (from 1 to 7) and the memory depth (from 1 to 5). The tests were repeated for all five predistorters that are the RTNTB predistorter, the EMP predistorter, and the proposed predistorter with the three sampling rates (128.88 MSa/s, 61.44 MSa/s, and 49.15 MSa/s). For each size, the corresponding predistorter was used to linearize the power amplifier, and the adjacent channel leakage ratio was measured in the adjacent channel and alternate adjacent channel. Here, the alternate adjacent channel refers to the next channel beyond the adjacent channel.

**Figure 15:**
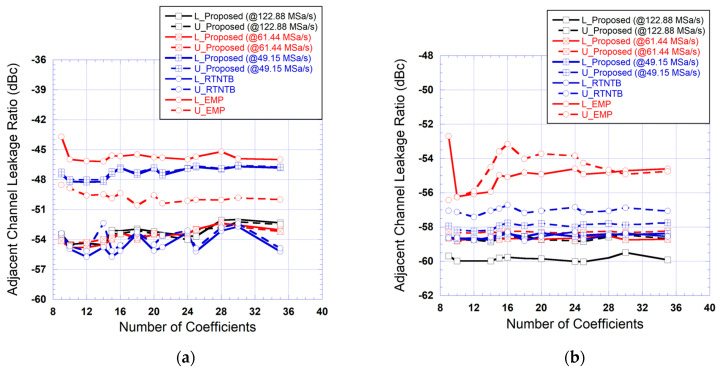
Measured adjacent channel leakage ratio at the output of the linearized amplifier as a function of the number of coefficients in the memory polynomial function for the benchmark digital predistorters and the proposed predistorter derived and implemented using various reduced sampling rates (Case I: 20 MHz test signal): (**a**) ACLR in the adjacent channel; (**b**) ACLR in the alternate adjacent channel.

The results of these tests are summarized in Figure 15, which presents the measured ACLR in the adjacent channel and alternate adjacent channel as a function of the number of coefficients in the memory polynomial function of the predistorter. In some cases, more than one combination of nonlinearity order and memory depth can lead to the same total number of coefficients. For these cases, the reported results are the ones corresponding to the combination that leads to the best ACLR. These results show that the proposed predistorter performance is not affected by a 50% reduction in the sampling rate (from 122.88 MSa/s to 61.44 MSa/s) in the forward and feedback paths. Moreover, for these two sampling rates, the performances of the proposed predistorter are comparable to what is obtained using the RTNTB predistorter derived at 122.88 MSa/s. However, a further reduction in the sampling rate (to 49.15 MSa/s) leads to significant degradation in the performance of the proposed predistorter. This figure also shows that the EMP-based predistorter performances are lower than that of the RTNTB-based predistorter and the proposed predistorter (derived at 122.88 MSa/s and 61.44 MSa/s) for the considered range of coefficients. The EMP performance is characterized by a noticeable imbalance between the upper and lower ACLR channels. It is important to note here that while the performance of the proposed predistorter shows a degradation when the sampling rate of the forward and feedback paths are reduced by 60%, the obtained results are still compliant with the 5G standard requirements for adjacent channels emissions as they are better than −45 dBc.

### 4.3. Case II—Validation Using 40 MHz Test Signal

**Figure 16:**
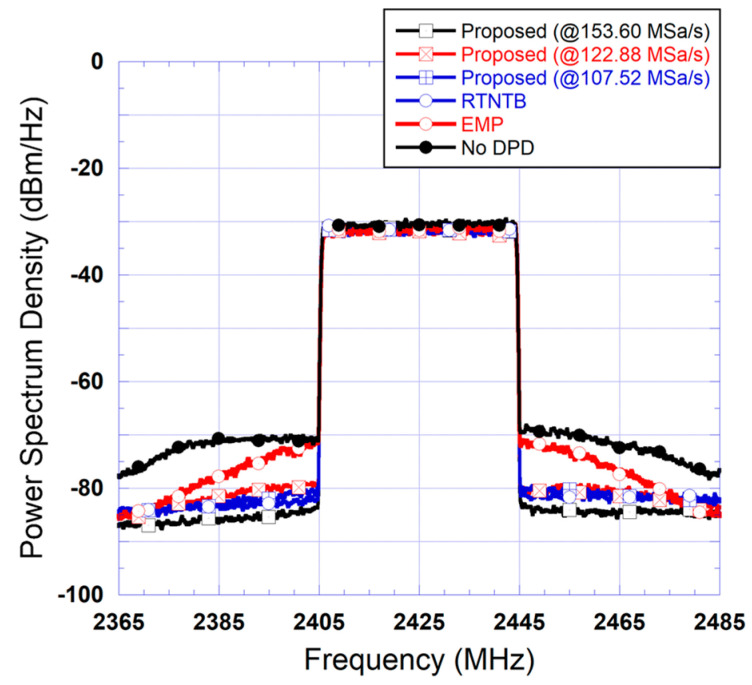
Measured spectra at the output of the linearized amplifier using benchmark digital predistorters and the proposed predistorter derived and implemented using various reduced sampling rates (Case II: 40 MHz test signal).

The same tests performed in the case of the 20 MHz test signal were repeated for the 40 MHz test signal. First, the proposed predistorter was derived at various sampling rates and then used to linearize the power amplifier driven by the 40 MHz 5G signal. The two benchmark predistorters were also applied to linearize the power amplifier operating under these conditions. The spectra measured at the output of the linearized power amplifier are reported in Figure 16. This figure also includes the spectrum measured at the output of the power amplifier before linearization. The results reported in this figure correspond to predistorters where the polynomial function has 15 coefficients corresponding to a nonlinearity order of 5 and a memory depth of 2.

**Figure 17:**
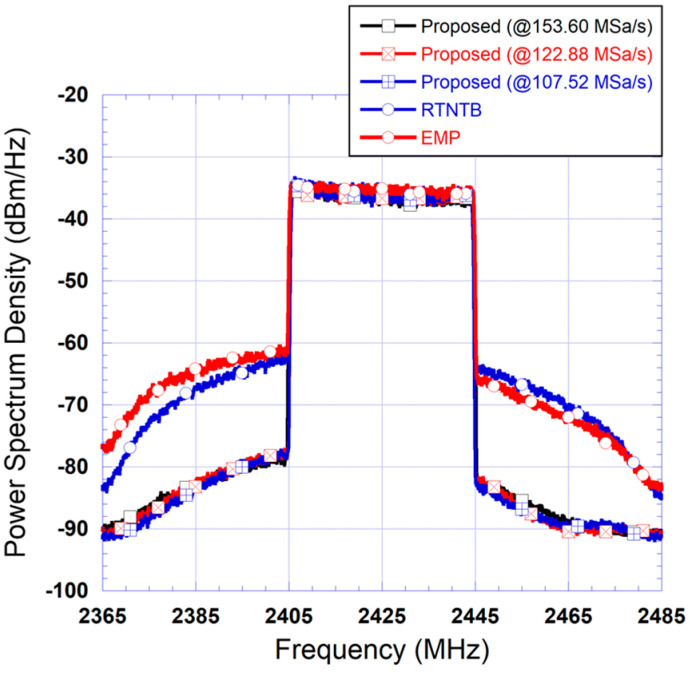
Measured spectra at the input of the digital-to-analog converter in the signal forward path for the benchmark digital predistorters and the proposed predistorter derived and implemented using various reduced sampling rates (Case II: 40 MHz test signal).

First, the results of Figure 16 show that the EMP predistorter fails to enhance the linearity of the power amplifier and achieves very limited improvement. This is interesting since this model is the only potential candidate for analog predistortion with memory effects compensation. Moreover, this figure shows that the performance of the proposed predistorter is similar to that of the RTNTB benchmark for sampling rate reductions of up to 30% in both the forward and feedback paths. In the experimental validation, it was observed that a further reduction in the sampling rate of the forward and feedback paths results in performance degradation for the proposed predistorter. To further investigate this, the spectra of the signals at the input of the forward path’s digital-to-analog converter were derived and are reported in Figure 17. This figure shows that the use of the proposed predistorter leads to a significant reduction in the spectrum regrowth of the signal fed into the digital-to-analog converter. Moreover, comparing the spectra in this figure to its equivalent for the 20 MHz signal (Figure 14), it appears that the spectrum regrowth in the case of the 40 MHz signal is more pronounced with a significant imbalance. This can be attributed to the stronger memory effects observed when the power amplifier is driven by the second test signal having wider bandwidth. This pronounced spectrum regrowth unavoidably impacts the maximum reduction in the sampling rate, which can be used while maintaining satisfactory linearization capability.

**Figure 18:**
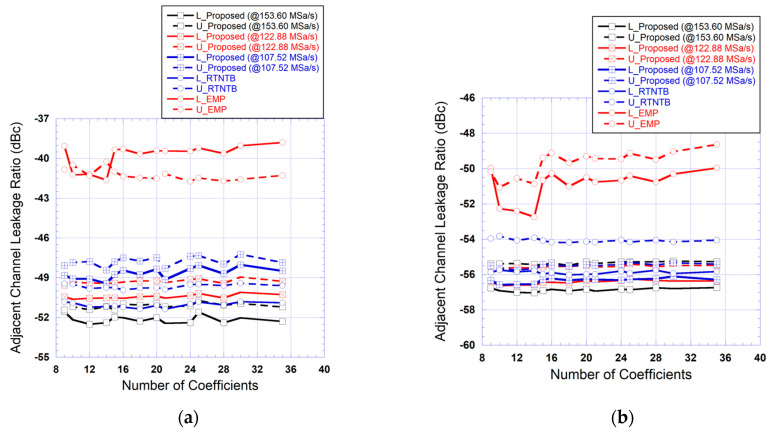
Measured adjacent channel leakage ratio at the output of the linearized amplifier as a function of the number of coefficients in the memory polynomial function for the benchmark digital predistorters and the proposed predistorter derived and implemented using various reduced sampling rates (Case II: 40 MHz test signal): (**a**) ACLR in the adjacent channel; (**b**) ACLR in the alternate adjacent channel.

The results of the ACLR measured at the output of the linearized amplifier while using various predistortion function settings are reported in Figure 18. These measurements were performed for the different number of coefficients in the memory polynomial sub-function of the predistorter. These results show that the EMP-based predistorter performance is consistently a few dBs worse than the other predistorters independently of the number of coefficients. Moreover, the performance of the proposed predistorter is comparable to that of the RTNTB benchmark. Most importantly, even when the sampling rate in the forward and feedback paths was reduced from 153.60 MSa/s to 107.52 MSa/s, the performance of the proposed predistorter was unaffected and remained within 2 dBs from that of the RTNTB benchmark.

## 5. Conclusions

In this work, a hybrid digital/analog predistorter was presented. The motivation behind this was to facilitate lower sampling rates in both the forward and feedback paths in preparation for next-generation wireless communication infrastructure development. The proposed hybrid predistorter is composed of a memory polynomial function implemented in the digital domain, and a memoryless nonlinear function in the analog domain. The memoryless nonlinear function linearizes a significant portion of the memoryless nonlinear behavior such that the forward DAC is required to linearize a much smaller bandwidth. Furthermore, experimental validation was performed for two cases in which a 20 MHz and 40 MHz bandwidth signals were utilized. The results of the first case study with the 20 MHz bandwidth signal indicates a possible reduction in sampling rate in both the forward and feedback paths by 60% when compared to the standard RTNTB model. Although the results showed some noticeable linearization degradation as the sampling rates were reduced, the linearization performance still remains within the standard requirements for spectrum emission mask. Furthermore, the second case study with the 40 MHz signal shows a similar trend with a sampling rate commensurate to 3×BW in contrast with the typical 5×BW. A major advantage of the proposed predistortion scheme is that a consistent reduced sampling rate is adopted in both the forward and the feedback paths, hence, reducing the computational complexity needed when different sampling rates are used in these paths.

## Figures and Tables

**Table 1:** Summary of key features of digital predistortion systems, analog predistortion systems, and the proposed hybrid predistortion system.

Feature	Digital Predistortion System	Analog Predistortion System	Proposed Hybrid Predistortion System
Sampling rate in the forward path	High (✗)	Moderate (✓)	Moderate (✓)
Sampling rate in the feedback path	High (✗)	High (✗)	Moderate (✓)
Feedback path complexity	Moderate (✓)	High (✗)	Moderate (✓)
Predistortion function accuracy	High (✓)	Limited (✗)	High (✓)
Predistortion function update	Simple (✓)	Complex (✗)	Simple (✓)
Linearization capability	High (✓)	Limited (✗)	High (✓)

**Table 2:** Summary of ACLR performance of the proposed predistorter, an APD and a DPD for the 20 MHz and the 40 MHz test signals.

ACLR			No Linearizer	APD Linearizer	DPD Linearizer	Proposed Linearizer
20 MHz	Adjacent Channel	ACLR_L	−32.5	−44.0	−55.4	−56.2
		ACLR_U	−32.5	−46.8	−55.1	−56.1
	Alternate Adjacent Channel	ACLR_L	−51.7	−59.4	−59.4	−60.3
		ACLR_U	−51.2	−58.8	−57.8	−59.2
40 MHz	Adjacent Channel	ACLR_L	−32.3	−43.2	−49.5	−50.7
		ACLR_U	−31.9	−46.7	−50.2	−51.6
	Alternate Adjacent Channel	ACLR_L	−51.6	−54.3	−53.5	−54.3
		ACLR_U	−50.9	−53.2	−52.7	−51.5

## Data Availability

Data are contained within the article.
